# From the Squat Rack to the Emergency Department: Internal Carotid Artery Dissection Following Heavy-Weight Squats

**DOI:** 10.7759/cureus.38139

**Published:** 2023-04-26

**Authors:** Endurance O Evbayekha, Anthony Willie, Foluke A Ogunlana, Kemar A Samuels, Ijeoma O Oaikhena, Adetoro T Okafor, Habiba I Ramon-Yusuf, Okelue E Okobi

**Affiliations:** 1 Internal Medicine, St. Luke’s Hospital, Chesterfield, USA; 2 Emergency Medicine, Igbinedion University, Okada, Benin City, NGA; 3 Family Medicine/General Practice, National Health Service Midlands, Derby, GBR; 4 Internal Medicine, Escuela Latinoamericana de Medicina, Kingston, JAM; 5 Health Sciences, Sault College, Sault Ste. Marie, CAN; 6 Epidemiology and Public Health, University of Minnesota School of Public Health, Minneapolis, USA; 7 Emergency Medicine, University Hospital Lewisham, London, GBR; 8 Family Medicine, Medficient Health Systems, Laurel, USA; 9 Family Medicine, Lakeside Medical Center, Belle Glade, USA

**Keywords:** non-traumatic carotid artery dissection, carotid artery occlusion, extracranial carotid dissection, dissection, carotid

## Abstract

Much is known about the implications of carotid artery dissection (ICAD), especially in the elderly population with abundant risk factors. However, the burden of ICAD in the young population is not extensively studied, and data in this area are few and far between. We present the case of a healthy American male who presented to the emergency department following visual disturbances that started at the gym a few hours before the presentation.

## Introduction

There is a growing awareness of the need for physical exercise, especially resistance training and weightlifting, because of the benefits of growing muscles on longevity and overall improved metabolism. There are no clear guidelines regarding how much weight is healthy for non-competing athletes in the gym. Weightlifting is a popular form of exercise in the United States and is practiced by people of various ages and fitness levels. Overall, weightlifting is a versatile and effective exercise that can benefit people of all ages and fitness levels, provided it is done safely and with proper technique. According to a 2011 to 2015 US Bureau of Labor survey and statistics on sports and exercise among Americans, weightlifting is the country’s second most popular fitness activity, with around 31.4 million Americans lifting weights regularly [1]. Weightlifting is popular among men and women, with men more likely to engage in heavy lifting and women more likely to focus on toning and sculpting. It is also popular among a wide range of age groups, from teenagers to seniors. People lift weights for various reasons, including improving muscle strength and size, increasing bone density, enhancing athletic performance, boosting metabolism and weight loss, and improving overall health and wellness. Some people also lift weights for bodybuilding or powerlifting competitions [1].

Internal carotid artery dissection (ICAD) is reportedly a rare condition. Some studies have demonstrated a 1.72 in 100,000 annual incidence [2]. Despite this, it is the most common cause of stroke in the young, accounting for about 23% of strokes in individuals under 50 [2-4]. There have been reports linking ICAD with weightlifting or intense exercises, although the exact relationship between these activities and ICAD is not well understood [5-7]. This rare but potentially life-threatening condition occurs when there is a tear in the inner lining of the carotid artery. This tear can lead to the formation of a blood clot that can cause a stroke or transient ischemic attack (TIA) [8]. Saw et al. conducted a prospective study on a Canadian cohort of 750 patients to investigate the natural course of spontaneous carotid artery dissection (SCAD). They found that approximately 28.9% of the observed population who engaged in weightlifting (>50 lbs) experienced SCAD [9]. Other rare occurrences linking intense physical activity, weightlifting, and ICAD have also been reported. In a case series of two cases by Dharmasaroja et al. [10], possible causative mechanisms and associations were described for sports-related ICAD. Despite the rarity of these cases, they have been documented, and efforts to comprehend the exact mechanism are ongoing [3,4]. In this regard, we report the case of a healthy young Asian American male who experienced an ICAD during a 130 kg squat session.

## Case presentation

A 27-year-old Asian American male with no significant medical history presented with right visual abnormality/loss. The patient was at the gym squatting when he first noticed a patchy visual loss in the right eye. He described it as seeing dark spots in front of the right eye; it was painless, intermittent, worsened by each squat session, and partially relieved with rest.

He denied headaches, fever, eye pain, or floaters. Notably, he had been squatting regularly with weights heavier than 130 kg for about four years without any problems. On examination, he was conscious, alert, and oriented to time, place, and person. There were no focal weakness or lateralizing signs, with no signs of meningism or cranial nerve dysfunction. Pupillary examination revealed mild miosis in the right eye, without ptosis. The patient’s sensation and deep tendon reflexes were unimpaired. Additionally, the results of tests such as gait analysis, finger-to-nose examination, Babinski sign evaluation, and Romberg test were all normal. His National Institute of Health Stroke Scale score was 0.

The vitals and physical examination were unremarkable except for a blood pressure (BP) of 159/104 mmHg. Laboratory examinations were essentially within the normal range, as shown in Table 1.

**Table 1:** Laboratory findings at presentation.

Laboratory test	Test result	Reference range
Complete blood count		
Hemoglobin	16.3	11.5–17 g/dL
Red blood cell count	5.1	4.1–5.6 × 10^6^/µL
Packed cell volume	49	35–50%
Mean copuscular volume	94	80–98 fL
Mean corpuscular hemoglobin	33.4	27–34 pg
Mean corpuscular hemoglobin concentration	35.2	32–36 g/dL
Red cell distribution width	14.3	11.8–15.0%
Platelets	283	140–420 × 10^3^/µL
Total white blood cell count	6.2	4–10.5 × 10^3^/µL
Complete metabolic panel		
Glucose, serum	89	62–99 mg/dL
Calcium, total, serum	9.3	8.7–10.3 mg/dL
Sodium	145	134–145 mmol/dL
Potassium	4.6	3.5–5.1 mmol/dL
Chloride	102	96–106 mmol/dL
Carbon dioxide, total	19	18–29 mmol/L
Albumin	4.2	3.5–4.7 g/dL
Total protein	8.0	6.0–8.4 g/dL
Alkaline phosphate	48	39–117 IU/L
Alanine transaminase	31	0–32 IU/L
Aspartate aminotransaminase	36	0–40 IU/L
Bilirubin	1.0	0–1.2 mg/dL
Blood urea nitrogen	21	8–27 mg/dL
Creatinine, serum	0.9	0.58–1.0 mg/dL
Blood urea nitrogen/Creatinine ratio	23	12–27
Others		
Thyroid-stimulating hormone	1.1	0.45–4.5 uIU/mL
Glycated hemoglobin	5.4	<5.7% = normal, 5.7–6.4% = prediabetes, and 6.5% or more = diabetes. Within 5.7–6.4% = prediabetes range
Erythrocyte sedimentation rate	20	0–27 mm/hour
C-reactive protein	2.4	<3 mg/L
Bilirubin	Negative	
Prothrombin time	1.0	0.9–1.1
International normalization ratio	13.6	11.9–14.9

**Figure 1:**
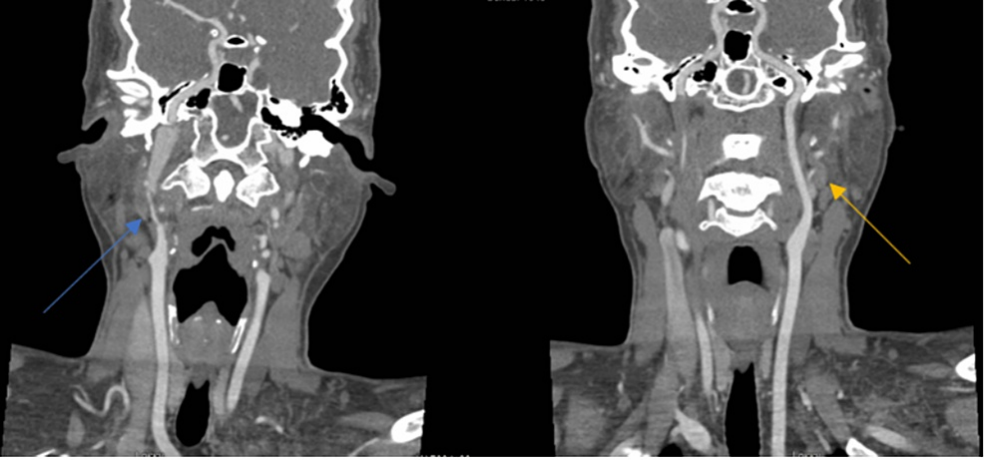
Computed tomography angiogram of the head and neck. The blue arrow represents the severe irregular stenosis of the cervical right internal carotid artery from shortly distal to its origin to its entering into the petrous portion with near-complete occlusion at the skull base. The yellow arrow shows the intact left carotid.

A computed tomography angiogram (CTA) of the head and neck in the emergency room showed right ICAD with near occlusion at the skull base (Figure 1).

**Figure 2:**
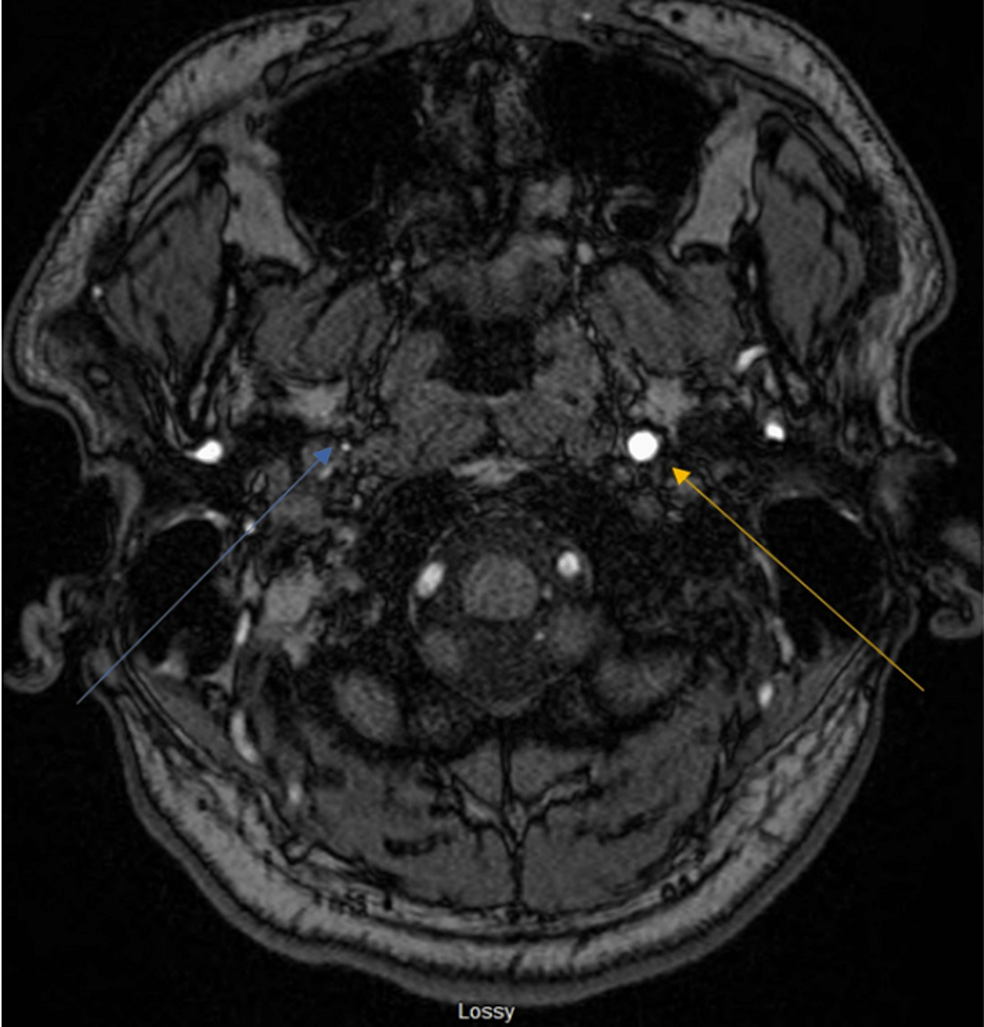
Magnetic resonance imaging of the brain. The blue arrow indicates dissection without flap and clotted blood completely filling the false lumen at the skull base versus intramural hematoma with critical narrowing at the skull base. The yellow arrow reveals normal anatomy.

**Figure 3:**
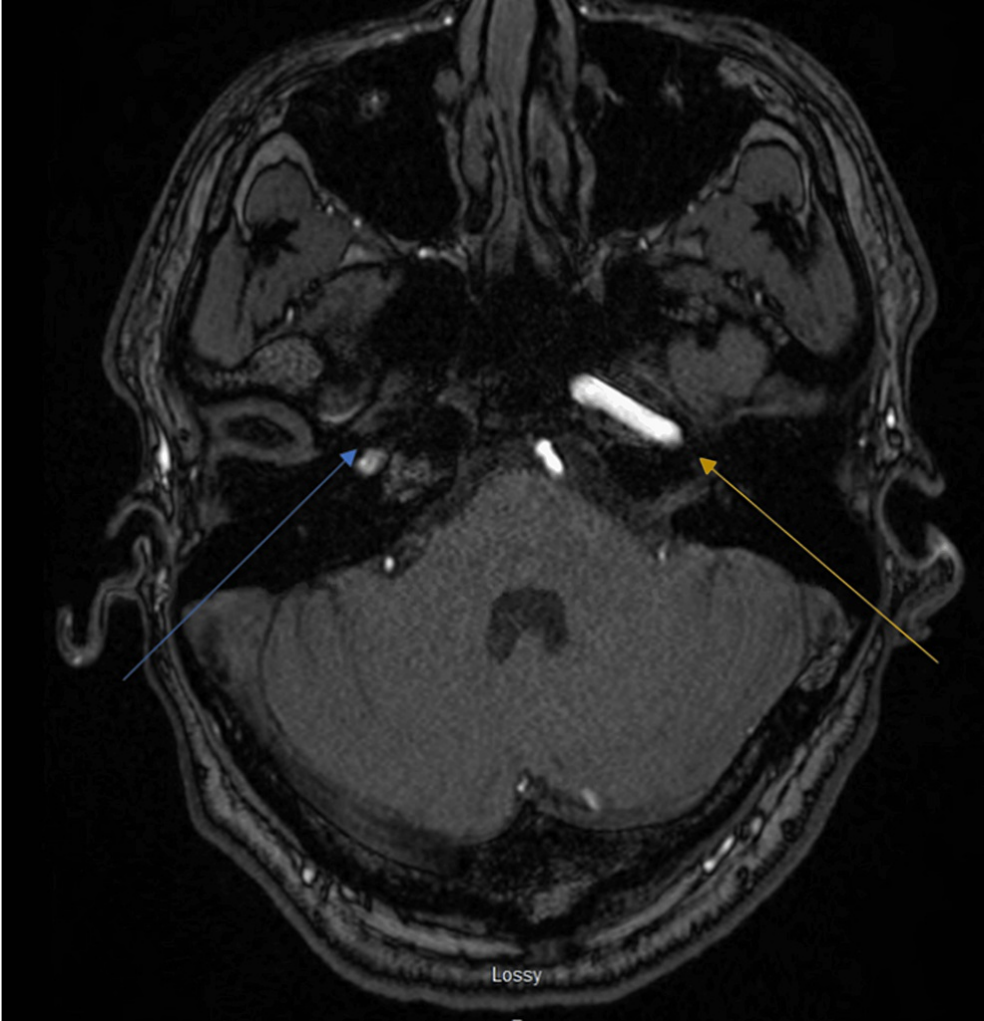
Magnetic resonance imaging of the brain. The blue arrows show very diminutive flow in the right internal carotid artery just above its origin. Diminutive signal but some flow in the right petrous internal carotid artery and distal internal carotid artery can be seen, suspected with retrograde ophthalmic artery. Some flow can also be noted in the right M1 and M2 middle cerebral arteries without any obvious large vessel clots.

A magnetic resonance imaging of the brain further revealed either a dissection without a flap and clotted blood completely filling the false lumen at the skull base or intramural hematoma with critical narrowing at the skull base. There was very diminutive flow in the right internal carotid artery just above its origin. There was a diminutive signal but some flow in the right petrous internal carotid artery and distal internal carotid artery, suspected with retrograde ophthalmic artery. Some flow was also noted in the right M1 and M2 middle cerebral arteries without any obvious large vessel clots (Figures 2, 3).

Electrocardiogram showed sinus rhythm. Echocardiography with agitated saline showed normal cardiac function and an ejection fraction of 53%. Urine toxicology was negative, and the patient denied the recreational use of steroids.

Hospital course

The patient’s BP continued to trend upwards of 170s/100s. He then received a dose of intravenous (IV) hydralazine in the ED. He was immediately started on IV unfractionated heparin. He also received 325 mg of aspirin. He was transferred to the cardiovascular intensive care unit, and the heparin drip was continued. Nicardipine drip was also initiated due to the uptrending BP, and the BP goal was to keep the systolic pressure below 140 mmHg.

Following evaluation and consensus between the neurologist and vascular surgeon, it was decided to transition the patient from heparin drip to clopidogrel, and oral antihypertensives amlodipine and metoprolol were initiated. After 36 hours of admission, he was discharged home to follow up with the vascular surgeon and cardiologist as an outpatient.

## Discussion

Like our patient, who was 27 years old, other researchers have found that ICAD is more prevalent in men but usually occurs in their 40s. Various risk factors have been linked to the condition, including factors that weaken the intima of arterial walls, such as smoking, high BP, diabetes, vascular disease, connective tissue disease, neck manipulation, and blunt trauma.

Despite previous reports, the precise mechanism behind heavy weightlifting causing ICAD has not been clearly understood. However, some observational studies have documented the elevation of BP with each active lift as a likely explanation [1,3]. In these observational studies, the authors reported incremental elevations of BP with repetitive lifts that might be compounded by pressure from Valsalva maneuvers, suggesting that intense pressure elevations leading to intimal wall tear could be a possible explanation.

ICAD is more prevalent during the winter, in contrast to our case. Numerous risk factors are linked with ICAD, with fibromuscular dysplasia being the most prevalent. Our patient’s high BP at the time of diagnosis was determined to be a risk factor for ICAD. Patients with ICAD may exhibit vague symptoms, but over 50% may also experience localized neurological symptoms caused by retinal or cerebral ischemia, such as Horner syndrome or cranial nerve paralysis. While many patients may not show any symptoms, it is crucial to maintain a high level of suspicion when risk factors are present to prevent additional complications [4]. Our patient displayed a classic symptom known as amaurosis fugax, characterized by temporary episodes of blindness caused by diminished blood flow to the retina.

In the presence of a high index of suspicion, CTA and magnet resonance angiography (MRA) of the head and neck are the preferred diagnostic modalities if the patient’s renal status is within the safe range for the radiological contrast. Some institutional protocols may prefer MRA when ICAD is suspected, with mural expansion and intramural blood flow as hallmarks for ICAD. In some other centers, CTA has demonstrated 100% sensitivity in diagnosing ICAD with principal features, including soft tissue swellings and changes in the caliber of the vessel or hematoma around the carotid artery [8]. Extracranial ICAD is more common than intracranial [11], as seen in our patient. Like in our patient, it usually occurs 2-3 cm superior to the bifurcation of the carotid artery [10]. The pathophysiology of artery dissection is due to an abnormality in the carotid artery’s vascular layers, resulting in blood seeping into a false lumen and creating an initial flap closing off distal circulation [2-4]. As seen in this patient, a clear radiological indicator of a carotid artery dissection is the presence of an intimal flap and double lumen, which occurs due to the formation of an intramural hematoma. Additional tests that may be considered include ultrasonography of the neck, echocardiography, electrocardiography, neuroimaging such as contrast and non-contrast CT or MRI of the brain, and electroencephalography.

Similar to this index patient, the risk-benefit ratio of anticoagulants is assessed upon diagnosis. There is currently no consensus on the optimal surgical management of ICAD, as it may vary depending on factors such as the patient’s clinical or hemodynamic state, comorbidities, or the mechanism of injury. A case-by-case approach has been documented in several publications detailing the management of weightlifting-associated injuries [8].

The administration of IV heparin, commonly regarded as an effective medical intervention to prevent thromboembolic complications, was commenced after identifying a thrombus to prevent further neurological deficits. A considerable proportion of patients with ICAD demonstrate favorable prognostic outcomes, marked by normal recanalization of the artery or alleviation of stenotic symptoms, resulting in complete recovery [12-14].

## Conclusions

Although ICAD is rare, it is the most common cause of stroke in the young. Although the exact mechanism that led to this occurrence in our patient is not fully understood, this case adds to the growing body of evidence indicating that intense physical activity, particularly weightlifting, maybe a potential risk factor for ICAD. Utilizing annual clinic appointments presents an opportunity to assess BP levels and educate young adults, particularly those enthusiastic about extreme bodybuilding and weightlifting, about the potential advantages and risks associated with intense weight training. However, there is a need for more extensive research to provide a reliable, evidence-based approach in this area.
